# Ablation of myocardial autonomic ganglion plexus in the treatment of bradyarrhythmia A one-arm interventional study

**DOI:** 10.1016/j.clinsp.2024.100448

**Published:** 2024-08-02

**Authors:** Mingliang Shao, Chenhuan Yao, Yafan Han, Xianhui Zhou, Yanmei Lu, Ling Zhang, Yaodong Li, Baopeng Tang

**Affiliations:** a Department of Cardiovascular, The People's Hospital of Xuancheng City, Xuancheng City, Anhui Province, China; b Department of Pacing Electrophysiology, Xinjiang Key Laboratory of Electrophysiology and Cardiac Remodeling, The First Affiliated Hospital of Xinjiang Medical University, Urumqi City, Xinjiang Uygur Autonomous Region, China; c Department of Research and Teaching, The People's Hospital of Xuancheng City, Xuancheng City, Anhui Province, China

**Keywords:** Ganglion plexus, 3D mapping, Ablation, Bradyarrhythmia, Interventional study

## Abstract

**Objectives:**

To study the complications and effectiveness of the treatment of chronic arrhythmias with cardiac Ganglion Plexus (GP) ablation, and to explore the value of the treatment of chronic arrhythmias with GP ablation.

**Methods:**

This study was a one-arm interventional study of patients from the first hospital of Xinjiang Medical University and the People's Hospital of Xuancheng City admitted (09/2018-08/2021) because of bradyarrhythmia. The left atrium was modeled using the Carto3 mapping system. The ablation endpoint was the absence of a vagal response under anatomically localized and high-frequency stimulation guidance. Postoperative routine follow-up was conducted. Holter data at 3-, 6-, and 12-months were recorded.

**Results:**

Fifty patients (25 male, mean age 33.16 ± 7.89 years) were induced vagal response by either LSGP, LIGP, RAGP, or RIGP. The heart rate was stable at 76 bpm, SNRT 1.092s. DC, DR, HR, SDNN, RMSSD values were lower than that before ablation. AC, SSR, TH values were higher than those before ablation, mean heart rate and the slowest heart rate were significantly increased. There were significant differences in follow-up data between the preoperative and postoperative periods (all p < 0.05). All the patients were successfully ablated, and their blood pressure decreased significantly. No complications such as vascular damage, vascular embolism and pericardial effusion occurred.

**Conclusions:**

Left Atrial GP ablation has good long-term clinical results and can be used as a treatment option for patients with bradyarrhythmia.

## Highlights

• SNRT 1.092s. DC, DR, HR, SDNN, RMSSD value are lower after ablation.
• AC, SSR, TH value were higher after ablation.
• No complications occurred after ablation.
• Left Atrial GP ablation has good long-term clinical results for bradyarrhythmia.

## Introduction

Ventricular arrhythmias are a group of arrhythmias characterized by wide complex QRS patterns, usually with rapid rates, resulting from an abnormal circuit or focus in the ventricles. Ventricular arrhythmias are most often due to underlying cardiac conditions (which may be secondary to reversible causes), and they are sometimes associated with cardiac arrest or sudden cardiac death if not treated immediately. Most cases of ventricular arrhythmias are tachyarrhythmia, but bradyarrhythmia also occurs.^1^ Bradyarrhythmia is a cardiac rhythm with appropriate cardiac contraction but resulting in a heart rate of < 60 bpm and can be caused by a wide variety of intrinsic and extrinsic etiologies.^2^ The prevalence of bradyarrhythmia is estimated at 1 per 600 adults >65 years of age, but epidemiological data are lacking. An abnormal increase of cardiac vagal tone will have a negative impact on the myocardium, suppress cardiac automaticity, excitability, and conductivity, and then lead to abnormal sinus node function and severe problems of atrioventricular conduction disorder, and finally cause symptomatic chronic arrhythmia. Patients generally have no obvious abnormalities in heart structure and function. Still, they often have shortness of breath, palpitation, dizziness, syncope, fatigue, and chest tightness, which affect labor and lead to a decline in quality of life.^2^^,^^3^

The efficacy of drugs for the management of bradyarrhythmia is challenging to determine, and compliance is usually poor. On the other hand, the use of a pacemaker has a relief effect on the condition.^4^ Still, it does solve the etiology, and the patients will have to replace the pacemaker many times in a lifetime. In addition, this treatment will lead to a variety of pacing-related complications. Hence, many young patients are reluctant to accept a pacemaker, affecting their prognosis.^5^

The use of cardiac ganglion plexus (Ganglionated Plexus, GP) ablation to treat bradyarrhythmia caused by high vagal tension has become a new treatment, bringing hope for the radical cure of atrioventricular block, paroxysmal sinoatrial node dysfunction, and vagal mediated syncope.^6^, ^7^, ^8^, ^9^, ^10^ This method is relatively novel, and data are still needed to determine its efficacy and safety and the approaches.

Therefore, the purpose of this study was to evaluate the complications and effectiveness of cardiac ganglion plexus ablation in the treatment of bradyarrhythmia by anatomical location combined with high-frequency stimulation of common ganglion sites around the left atrial endocardial pulmonary vein.

## Materials and methods

### Medical ethics

This study was approved by the Ethics Committee of the First Affiliated Hospital of Xinjiang Medical University (K201910-02) and registered with the China Clinical trial Registration Center (ChiCTR1900027305). The patients signed a written informed consent form before the operation. All clinical studies followed the ARRIVE guidelines.

### Patient data

This study was a one-arm interventional study. A total of 50 patients with chronic arrhythmia due to chronic cardiac arrhythmia and frequent symptoms such as shortness of breath, palpitations, dizziness, fainting, Crohn's, fatigue and chest tightness were admitted from September 2018 to August 2021 from the medical record system of the first hospital of Xinjiang Medical University and the People's Hospital of Xuancheng City. All patients were discontinued from all drugs before ablation therapy and underwent left atrial endocardial GP ablation with anatomical location combined with high-frequency stimulation under the guidance of Carto 3 (Johnson & Johnson, New Brunswick, NJ, USA).

### Inclusion and exclusion criteria

#### Inclusion criteria

1. All patients had bradyarrhythmia with clinical symptoms such as palpitations, dizziness, fainting, amaurosis, fatigue, chest tightness, and other symptoms.
2. Patients with positive atropine test for esophageal pacing.
3. 18‒50 years of age.
4. Patients who had the characteristics that showed that permanent pacemaker implantation was needed.
5. Routine treatments are ineffective, or the side effects cannot be tolerated.
6. Consented to the GP ablation of the left atrium and regular postoperative follow-up.

#### Exclusion criteria

Patients with sinus node illness, atrioventricular block, hypertrophic cardiomyopathy, pulmonary hypertension, epilepsy, transient ischemic attack, and subclavian theft syndrome.

1. Severe comorbidities, including myocardial infarction within 6 months, New York Heart Association heart function III‒IV, diabetes, or terminal illness.
2. History of cardiac surgery, catheter ablation, or permanent pacemaker implantation.
3. Patients who refused GP ablation or postoperative clinical follow-up.

### Preoperative preparation

All patients were examined for cardiovascular, neurological, and psychiatric disorders before the operation. Detailed medical history, physical examination, and electrocardiogram were performed. Conventional electrophysiological examination and evaluation of the sinoatrial node function, including sinus node recovery time, were performed to eliminate sick sinus syndrome and other potential cardiac arrhythmias. The general data of patients were collected, including physical examination, demographic characteristics, laboratory examination, electrocardiogram, echocardiography, dynamic electrocardiogram, esophageal pulsation, and current medication.

Patients were monitored using a 10-electrode 12-lead ambulatory electrocardiogram. Researchers and patients utilized a double-blind method throughout the Holter data analysis process to compare each R-R interval with the preceding cardiac cycle's R-R interval. A shorter R-R interval compared to the previous cycle was identified as an Acceleration Cycle (AC), whereas a longer interval was termed a Deceleration Cycle (DC). Each deceleration and acceleration point was assigned a corresponding number. The length of cardiac rhythm segments was determined based on the lowest heart rate and ranked accordingly. The DC or AC value was calculated from the average of these serial numbers.

### Identification of the GP

All patients received fentanyl intravenous sedation, analgesia, and anesthesia. The conventional Seldinger puncture of the left subclavian vein and the femoral vein was carried out. Coronary sinus and right ventricular electrodes were placed. Electrophysiological data were recorded using a model 64 recorder (GE Healthcare, Waukesha, WI, USA), with a filter bandwidth of 30‒500 Hz. Under X-Ray fluoroscopy, an interatrial septum needle was used to puncture the interatrial septum. Intravenous heparin anticoagulation was used to control the thrombin time within 200‒300 ms. Using Carto3 (Johnson & Johnson, New Brunswick, NJ, USA), a 4-mm ablation catheter (Johnson & Johnson, the United States) was advanced and guided by three-dimensional modeling for anatomic positioning and high-frequency stimulation to the GP.^6^^,^^11^, ^12^, ^13^ The main sites with dense distribution of autonomic ganglion are the Left upper Ganglion (LSGP), which is located in the intersection area between the left atrium and/or Left Atrial Appendage (LAA) of the left upper pulmonary vein, the Left Inferior Ganglionated Plexus (LIGP), which is located at the junction below the left inferior pulmonary vein orifice with the left atrium, the Right Anterior Ganglion (RAGP), which is located at the junction of the anterior wall of the right superior pulmonary vein and the left atrium, and the lower Right Ganglion (RIGP), which is located at the junction of inferior vena cava and left and right atrium.^8^ After the anatomic location was determined, the distal electrode of a 4-mm head ablation catheter was applied to the intima of the left atrium to perform high-frequency stimulation (HFS, 20 Hz, 10‒20 V, 5 ms) for localization of the GP with GE Healthcare Stimulation instrument (GE Healthcare, Waukesha, WI, USA).^14^^,^^15^ The filtering range of intracardiac electrical signals was set as 30‒500 Hz, and the measuring screen speed was 100 mm/s. Attention was paid to avoiding ventricular capture and ventricular tachycardia or fibrillation during high-frequency stimulation near the mitral annulus.^16^ When the heart rate slowed down with high-frequency stimulation, the location of the GP was marked in the three-dimensional model. During the operation, blood pressure, oxygen saturation, surface electrocardiogram, and endocardial bipolar potential were continuously monitored.

### Autonomic ganglion ablation

A Radiofrequency Ablation (EP Shuttle, Johnson & Johnson, New Brunswick, NJ, USA) was used for temperature-controlled radiofrequency ablation with a 4-mm ablation electrode under the guidance of the 3D mapping system after the positioning of the neural nodes. The upper-temperature limit was no higher than 45°C, and the upper power limit was less than 45W. Ablation was performed in accordance with the Left Superior Ganglionated Plexus (LSGP), Left Inferior Ganglionated Plexus (LIGP), Right Anterior Ganglionated Plexus (RAGP), and Right Inferior Ganglionated Plexus (RIGP), and the site of ablation was marked on the three-dimensional model of the left atrium. Transient ventricular arrest, a rapid decrease of blood pressure, atrioventricular block, or extension of R-R at least 50% immediately after high-frequency stimulation or ablation were defined as a positive vagal response, and the site was confirmed as an effective GP stimulation site 18. All ablation sites were ablated until the vagal reaction disappeared, and each discharge was more than 60. The criteria to judge the success of ablation were as follows: the maximum voltage stimulated GPs site, which would not cause significantly slower heart rate or significantly delayed atrioventricular node conduction, and no vagal reaction occurred in all GPs. In addition, standby right ventricular pacing was required during the operation to prevent a vagal response and the negative effect of ventricular arrest. Baseline indicators such as heart rate and AH interval were recorded during the whole process.

### Follow-up

All patients received no other drug therapy except aspirin for 1-month after the operation. The patients were followed for 3-, 6-, and 12-months after the operation. Echocardiography (ECG), 12-lead electrocardiogram, and 24-hour dynamic electrocardiogram (Holter) were performed at each follow-up. The Holter data at 3-, 6-, and 12-months after ablation were recorded to analyze the changes in heart rate and time-domain variability before and after ablation.

### Statistical analysis

The data for normally distributed variables were represented as mean ± standard deviation. Paired samples test was used to analyze the changes in Deceleration Runs (DRs) values, Systolic Strain Rate (SSR) values and Tyrosine Hydroxylase (TH) values after the ablation procedure. Repeated-measures ANOVA was carried out to determine whether significant changes in Heart Rate (HR) values, the standard deviation of Normal-to-Normal Intervals (SDNN) values, Root mean square successive differences between successive R-R intervals (RMSSD) values, Deceleration Capacity (DC) values and heart rate Acceleration (AC) values over the course of 12-months after the procedure. Two-sided p < 0.05 were considered statistically significant. All analyses were carried out using SPSS 25.0 (SPSS Inc, Chicago, USA).

## Results

### Baseline data and ablation results

This study comprised 50 patients (25 males and 25 females, mean age 33.16 ± 7.86), ranging from 18 to 51 years. Baseline characteristics of the patients are shown in Table 1.

**Table 1:** Baseline characteristics of the 50 patients.

Item	Value
Age, years (mean ± SD)	33.16 ± 7.89
Male, n (%)	25 (50)
Dizziness, n (%)	35 (70)
Amaurosis, n (%)	10 (20)
Syncope, n (%)	20 (40)
Palpitation, n (%)	20 (40)
Chest tightness, n (%)	10 (20)
Esophageal modulation	
SNRT max, ms (mean ± SD)	1224.12±324.32
CSNRT, ms (mean ± SD)	291.20±102.82
A-V Venturi point, bpm (mean ± SD)	122.90±21.88
Echocardiography	
LA, mm (mean ± SD)	31.90±1.04
LVDD, mm (mean ± SD)	46.20±2.67
LVEF (%)	63.41±2.22

CSNRT, Corrected Sinus Node Recovery Time; LA, Left Atrium; LVDD, Left Ventricular Diastolic Dysfunction; LVEF, Left Ventricular Ejection Fraction.

The ablation was performed 19.46 ± 6.25 times, with a discharge time of more than 60 seconds. The average ablation time was 7.98 ± 2.68 minutes, the exposure time of 6.20 ± 2.27 minutes, and the average total operation time was 48.16 ± 5.87 minutes.

**Fig. 1:**
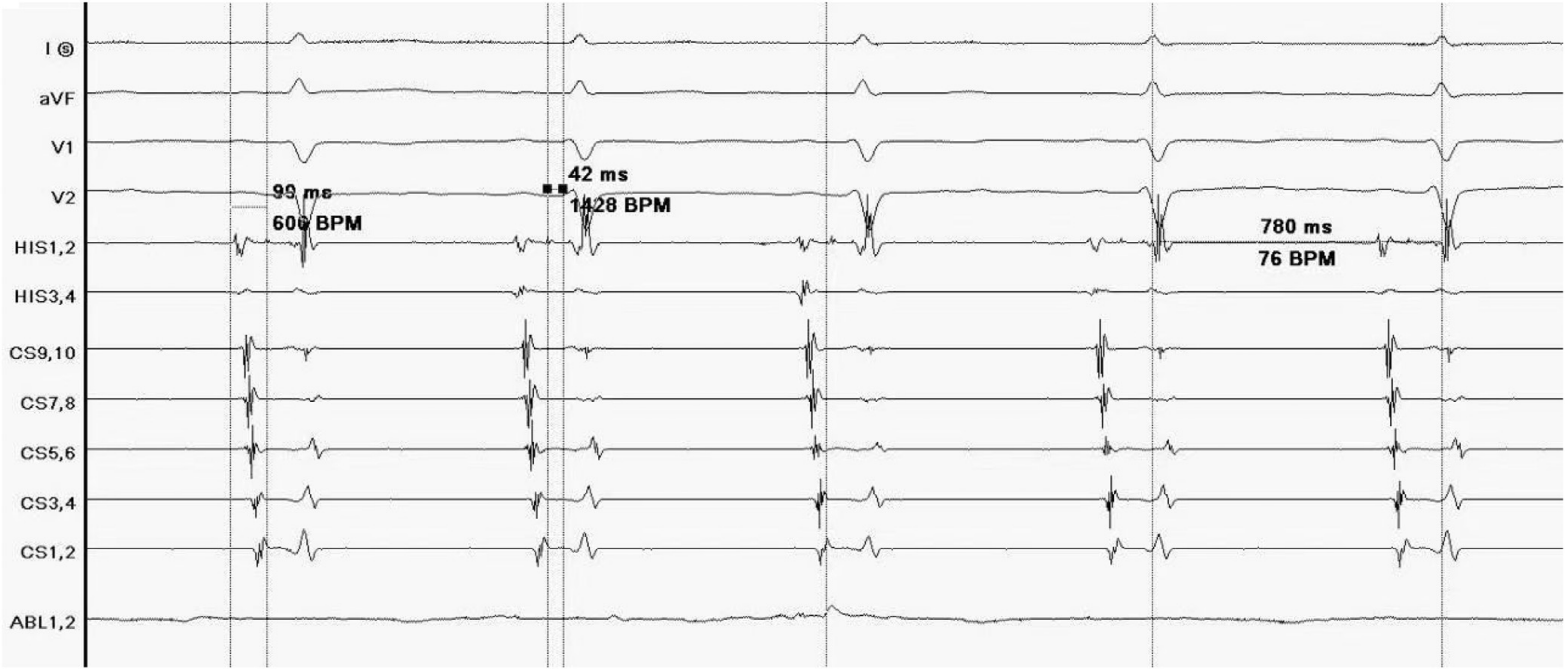
The heart rate was stable at 76 bpm.

**Fig. 2:**
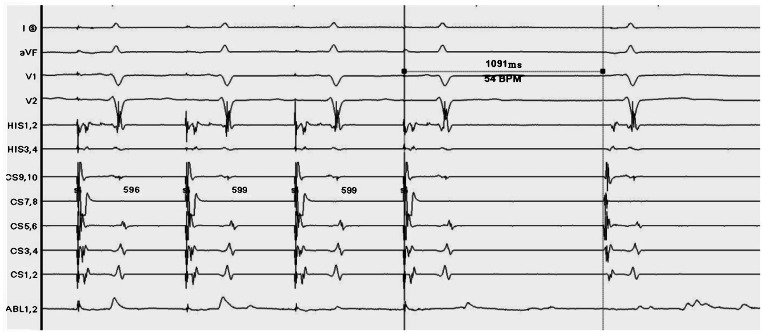
The sinoatrial node is functioning normally.

**Fig. 3:**
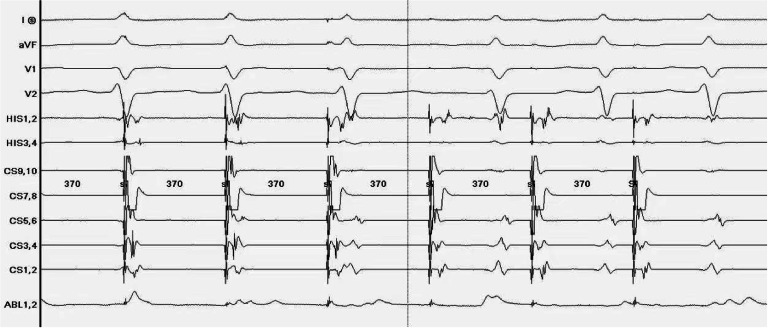
Pretransfer refractory period 370 ms.

During ablation, at least one of LSGP, LIGP, RAGP, and RIGP can be used to induce a vagal response. After ablation, blood pressure significantly decreases. After ablation of the posterior tip of the left superior pulmonary vein, the heart rate slowed to 44 bpm and a vagal reaction occurred. The anterior edge of the right upper lung was ablated, and the heart rate increased to 82 bpm. The heart rate accelerated, and the vagal response disappeared. After observation for 20 minutes, Sinus Node Recovery Time (SNRT) was 1.092 seconds, heart rate stabilized at 76 bpm (Fig. 1), and sinus node function returned to normal (Fig. 2). Pretransfer refractory period was 370 ms (Fig. 3). All patients underwent successful ablation without any complications such as vascular injury, thromboembolism, and pericardial effusion. During the follow-up period, none of the patients had any arrhythmia symptoms.

After ablation, DR2, DR4, DR8 values were lower than those before ablation, and SSR (amplitude), SSR (latent period), TH values were higher than those before ablation (Table 2).

**Table 2:** Comparison of detection indexes before and after ablation (χ ± s).

Item	DR2	DR4	DR8	SSR (amplitude)	SSR (latent period)	TH
Preoperative	8.36±1.37	0.81±0.28	0.09±0.01	1.94±1.19	1.56±0.22	26.75±9.15
Postoperative	7.32±1.35	0.45±0.19	0.06±0.01	1.05±0.74	1.49±0.35	16.17±7.24

Using SPSS software, a paired samples test was performed on DRs values, SSR values, and TH values before and after ablation. There were significant differences in these data between the preoperative and postoperative periods (all p < 0.05) (Table 3).

**Table 3:** Paired Samples Test.

Paired Samples Test									
		Paired Differences					t	df	Sig. (2-tailed)
		Mean	Std. Deviation	Std. Error Mean	95% Confidence Interval of the Difference				
					Lower	Upper			
Pair 1	DR2 before ablation ‒ DR2 after ablation	1.03980	0.36469	0.05157	0.93616	1.14344	20.161	49	0.000
Pair 2	DR4 before ablation ‒ DR4 after ablation	0.36240	0.12037	0.01702	0.32819	0.39661	21.290	49	0.000
Pair 3	DR8 before ablation ‒ DR8 after ablation	0.02880	0.00480	0.00068	0.02744	0.03016	42.444	49	0.000
Pair 4	SSR (amplitude) before ablation ‒ SSR (amplitude) after ablation	-0.88220	0.45443	0.06427	-1.01135	-0.75305	-13.727	49	0.000
Pair 5	SSR (latent period) before ablation – SSR (latent period) after ablation	-0.06020	0.13667	0.01933	-0.09904	-0.02136	-3.115	49	0.003
Pair 6	TH before ablation - TH after ablation	-10.5728	2.40434	0.34003	-11.25611	-9.88949	-31.094	49	0.000

Note: DR2 refers to the phenomenon of successive heart rate deceleration occurring in the last two cardiac cycles of three consecutive cardiac cycles. DR4 refers to the phenomenon of successive heart rate deceleration occurring in the last four cardiac cycles of five consecutive cardiac cycles. DR8 refers to the phenomenon of successive heart rate deceleration in the last eight cardiac cycles of nine consecutive cardiac cycles.

### Follow up results

Fifty patients were followed up for 12 months. The index (Max HR, Min HR, Mean HR, SDNN, RMSSD, DC, AC) at 3-, 6-, and 12-months after ablation was measured. The results showed that there was no significant difference in Max HR after ablation treatment compared to pre-treatment, whereas Min HR and Mean HR increased significantly after ablation treatment. The effect of Cardiac autonomic ganglia plexus association on subjects was evaluated using the repeated-measures ANOVA.

**Fig. 4:**
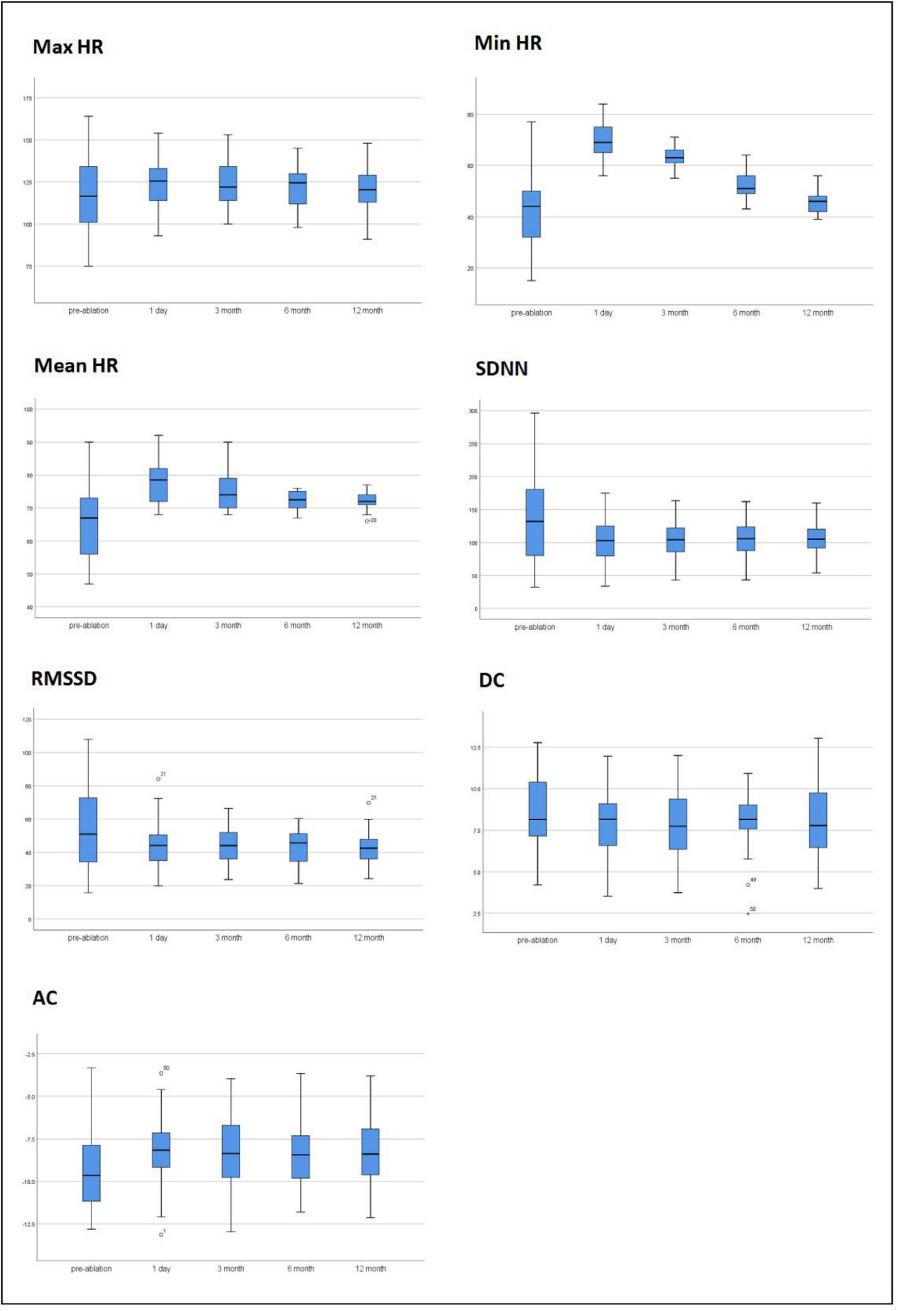
Boxplots results for Max HR, Min HR, Mean HR, SDNN, RMSSD, DC, AC value.

According to the boxplots (Fig. 4), there were no significant differences in the abnormal values of the data. According to the Shapiro-wilk test results, the data in each group followed a normal distribution (p > 0.05). According to Mauchly's test of spherical, the variance covariance matrices of the dependent variables were not equal and were corrected using the Epsilon (Greenhouse & Geisser) method.

**Fig. 5:**
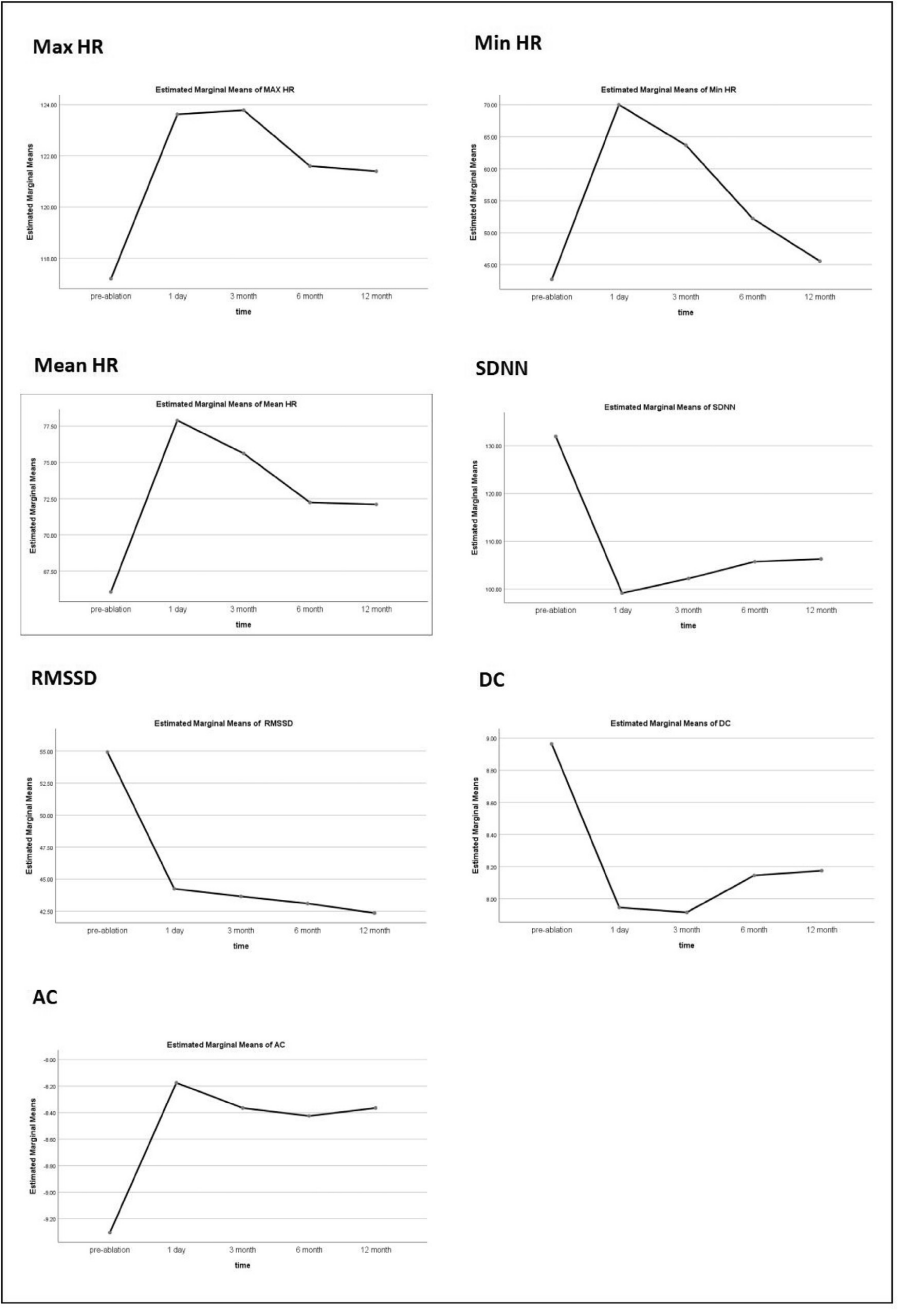
estimated marginal means outline graph results of Max HR, Min HR, Mean HR, SDNN, RMSSD, DC, AC value.

The data was expressed in the form of mean ± standard deviation, and the data before and after ablation were statistically significant. After correction, FMax HR = (1.777, 87.083) = 17.501, p = 0.000, FMin HR = (1.101, 53.955) = 379.730, p = 0.000, FMean HR = (1.342, 65.765) = 55.803, p = 0.000, FSDNN = (1.108, 54.312) = 12.118, p = 0.001, FRMSSD = (1.119, 54.813) = 11.520, p = 0.001, FDC = (1.995, 97.756) = 51.713, p = 0.000, FAC = (2.994, 146.690) = 144.812, p = 0.000. The specific analysis results are shown in Table 4. And then plotted an estimated marginal means outline graph based on the analysis results (Fig. 5).

**Table 4:** Results of repeated-measures ANOVA.

Item	Max HR (%)	Min HR (%)	Mean HR (%)	SDNN (ms)	RMSSD (ms)	DC (ms)	AC (ms)
Pre-ablation (χ ± *s*)	117.2±20.68	42.7±13.55	66.06±11.84	131.92±63.93	54.92±25.91	8.97±2.15	-9.31±2.06
Post-ablation (χ ± *s*)	123.62±13.86	70±6.12	77.9±6.22	99.13±31.02	44.25±13.49	7.95±2.02	-8.17±1.86
3-month (χ ± *s*)	123.78±12.66	63.68±4.15	75.62±5.85	102.19±26.50	43.65±10.50	7.91±1.99	-8.37±1.96
6-month (χ ± *s*)	121.6±12.38	52.2±4.82	72.24±2.78	105.74±23.54	43.10±10.70	8.15±1.62	-8.42±1.79
12-month (χ ± *s*)	121.4±12.39	45.56±3.86	72.1±2.30	106.30±20.52	42.35±9.70	8.18±2.11	-8.37±2.06
Corrected F	17.501	379.730	55.803	12.118	11.520	51.713	144.812
Corrected p	0.000	0.000	0.000	0.001	0.001	0.000	0.000
Partial Eta Squared	0.263	0.886	0.532	0.198	0.190	0.513	0.747

## Discussion

Drugs and pacemakers can be used to treat bradyarrhythmia, but they do not solve the etiology. GP ablation can be used to treat bradyarrhythmia, but it is a novel method. Therefore, this study aimed to evaluate the complications and effectiveness of cardiac GP ablation in the treatment of bradyarrhythmia. The results of this study indicate that GP ablation can be used in the treatment of chronic bradyarrhythmia's by using anatomical localization combined with high-frequency stimulation under the guidance of the Carto3 system, followed by high-intensity ablation of the left atrial endocardial ganglia with a large 4-mm electrode, which reduces the probability of autonomic nerve regeneration at the ablation site. In this pilot study, there was no recurrence after treatment with this strategy during the follow-up of 12 months. Similar results were obtained in patients with cardioinhibitory syncope and functional atrioventricular block. Nevertheless, according to the relevant reports after similar operations in patients with arrhythmia in general, there are indeed some postoperative recurrence cases, which might be caused by the ablation not reaching the ideal depth, the range being too small, and the ablation being too short.^17^, ^18^, ^19^, ^20^, ^21^, ^22^

The anatomical study of the cardiac autonomic nervous system showed that the left atrial autonomic ganglion was mainly distributed at the intersection of the left atrium and the perioral pulmonary vein.^7^ The LSGP is located at the intersection of the root of the Left Superior Pulmonary Vein (LSPV) and the left atria, extending to the middle of the left atria.^10^ The RAGP is located in front of the Right Upper Pulmonary Vein (RSPV) and the left atrium and extends downward.^7^ In addition, there are significantly fewer ganglia distributions in the Lower left (LIPV) and Right (RIPV) orifices than in the upper left and right pulmonary veins.^9^^,^^22^ In this study, the ganglion sites located by high-frequency stimulation were consistent with the anatomical structure. All patients showed vagal reflex (blood pressure and heart rate decreased) immediately after ablation at the location, and sinus heart rate increased after GP ablation.

A number of studies showed that the results of nerve ablation were inconsistent in the intermediate- and long-term.^19^ According to animal studies, cardiac autonomic nerve function can be restored within 4 weeks after the ablation of the fat pad. Still, the time of recovery of autonomic nerve function after human denervation ablation varies among individuals. Previous studies showed that the autonomic nerve function could be gradually restored after denervation. According to Scanavacca et al.,^10^ the autonomic nerve function of patients with atrial fibrillation after ablation is gradually restored from 3 to 6 months after surgery. Based on this conclusion, this study used a large 4-mm head and high power (45W) ablation to achieve the goal of extensive GP ablation of the left atrium. From the preliminary observation results, no complications occurred, indicating that this treatment method is possibly safe and reliable, but this will have to be confirmed in future trials.

DC is a newly discovered noninvasive Electrocardiogram (ECG) technique, which is used to evaluate the tension of the autonomic nerve. It can quantitatively evaluate the tension of the vagus nerve. The decrease in DC value indicates that the sensitivity of vagus nerve tension is weakened. This study found that the vagus nerve excitability in patients with bradyarrhythmia increased abnormally, the DC value decreased significantly after ablation, and the cardiac vagus nerve activity decreased. This treatment method has a significant effect.

The important information about cardiovascular regulation can be reflected by heart rate variation. The physiological basis of heart rate variability is attributed to the sympathetic and vagal nervous system. As an indirect quantitative evaluation of the myocardial sympathetic innervation, vagal tension and balance can be achieved by detailed analysis of heart rate variability. The vagal condition plays a decisive role in heart rate variability; therefore, the vagal nerve is hyperactive. This study suggests that GP ablation led to smaller heart rate variation, reduced SDNN and RMSSD, the vagal, enhanced sympathetic for the adjustment of the sinoatrial node function, and optimized sinoatrial node and atrioventricular node electrical physiological functions. Therefore, the heart rate increased in the patients. Besides, Holter at 3-, 6-, and 12-months after GP ablation showed the absence of disease recurrence in all patients. Nevertheless, long-term data are necessary.^23^^,^^24^

This study was a single-center, one-arm, non-randomized controlled study with a small number of patients. It is necessary to conduct large-sample randomized controlled trials to confirm the efficacy and safety of this treatment strategy and to conduct long-term follow-up. The current GP ablation target location and endpoint determination are not very accurate, and the technical means of potential neural recognition still needs to be accurately demonstrated by the technological progress in ganglion marker scanning, Magnetic Resonance Imaging (MRI), and other imaging auxiliary tools.^20^ At the same time, the postoperative follow-up period is long, and there are many external factors in the life of the patients that can influence heart rate, and it is difficult to eliminate objective risks through real-time monitoring.

## Conclusions

This pilot study suggests that GP ablation of the left atrial intima can improve sinus heart rate and alleviate discomfort in patients with symptomatic bradyarrhythmia. It is a potential treatment for bradyarrhythmia with the possibility of high safety and efficacy. Nevertheless, the long-term benefits of such treatment strategies require randomized, controlled studies based on a larger-scale sample to ensure in-depth validation of the conclusions.

## Data availability statement

Generated Statement: The raw data supporting the conclusions of this article will be made available by the authors, without undue reservation.

## Ethics statements

This study was approved by the Ethics Committee of the First Affiliated Hospital of Xinjiang Medical University (K201910-02) and registered with the China Clinical Trial Registration Center (ChiCTR1900027305). The patients signed a written informed consent form before the operation.

## Contribution to the field

This pilot study suggests that GP ablation of the left atrial intima can improve sinus heart rate and alleviate discomfort in patients with symptomatic bradyarrhythmia. It is a potential treatment for bradyarrhythmia with the possibility of high safety and efficacy.

## Studies involving animal subjects

Generated Statement: No animal studies are presented in this manuscript.

## Studies involving human subjects

Generated Statement: This study was approved by the Ethics Committee of the First Affiliated Hospital of Xinjiang Medical University (K201910-02) and registered with the China Clinical Trial Registration Center (ChiCTR1900027305). The patients signed a written informed consent form before the operation.

## Inclusion of identifiable human data

Generated Statement: No potentially identifiable human images or data is presented in this study.

## Authors’ contributions

Yaodong Li and Baopeng Tang conceived and designed the study and revised the manuscript for the study. Mingliang Shao, Chenhuan Yao and Yafan Han performed data analysis. Ling Zhang performed the data collection and collation. Xianhui Zhou and Yanmei Lu provided research guidance and revised the manuscript. The manuscript was written by Mingliang Shao and Chenhuan Yao. All authors contributed to and approved the submitted version of the manuscript.

## Funding

This work was supported by the Science Fund for Distinguished Young Scholars of Xinjiang Autonomous Region (n 2022D01E22); Key R&D Program of Xinjiang Uygur Autonomous Region (2022B03023) and the National Natural Science Foundation of China (n 81873487).

## Declaration of competing interest

The authors declare that the research was conducted in the absence of any commercial or financial relationships that could be construed as a potential conflict of interest.
